# Biomass estimation and characterization of the nutrient components of thinned unripe grapes in China and the global grape industries

**DOI:** 10.1016/j.fochx.2022.100363

**Published:** 2022-06-16

**Authors:** Mengyuan Wei, Tingting Ma, Muming Cao, Binsheng Wei, Chao Li, Caihong Li, Kekun Zhang, Yulin Fang, Xiangyu Sun

**Affiliations:** a College of Enology, College of Food Science and Engineering, Shaanxi Provincial Key Laboratory of Viti-Viniculture, Viti-viniculture Engineering Technology Center of State Forestry and Grassland Administration, Shaanxi Engineering Research Center for Viti-Viniculture, Heyang Viti-viniculture Station, Ningxia Eastern Foot of Helan Mountain Wine Station, Northwest A&F University, Yangling 712100, China; b Grape and Wine Research Institute, Guangxi Academy of Agricultural Sciences, Nanning 530007, China; c Shaanxi Zhangyu Ruina Castle Winery Co. LTD, Yantai Changyu Pioneer Wine Company Limited, Xi’an 710075, China; d Quality Standards and Testing Institute of Agricultural Technology, Ningxia Academy of Agricultural Sciences, Yinchuan 750002, China

**Keywords:** Biomass investigation, Biomass estimation, Thinned unripe grapes, Nutritional components, Functional components

## Abstract

The biomass of thinned unripe grape (TUR) was investigated and estimated in China and the world. In addition, the physicochemical parameters, nutritional and functional components and antioxidant activity of nine TUR and ripe grape fruit (RGF) samples were determined and analyzed. The results showed that about 1695.75 kt TUR was produced in China and as much as 14436.16 kt worldwide, which was closely related to the fruit thinning time. The total sugar and protein contents of TUR were significantly lower than those of RGF (*p* < 0.05), while the organic acids (especially tartaric acid and malic acid) and crude fiber of TUR were significantly higher than those of RGF (*p* < 0.05). Moreover, the total polyphenol, flavonoid, tannin and flavan-3-ols contents of TUR were 4.2–13.5, 3.6–12.3, 4.3–62.8 and 1.5–7.6 times those of RGF, respectively. Meanwhile, the antioxidant capacity of TUR was significantly higher than that of RGF, as well (*p* < 0.05). This study aimed to conduct in-depth research into the nutritional characteristics of TUR, propose the targeted direction for their further investigation and then lay a theoretical foundation from which the research findings could be applied in practice.

## Highlights

• Thinned unripe grape (TUR) biomass was investigated and estimated for the first time.
• Nutrition analysis was carried out on 9 TUR for the first time.
• About 1695.6 kt TUR can be produced in China yearly, and as high as 14436.2 kt worldwide.
• TUR is a rich source of phenols and organic acids.
• TUR may be a potential natural antioxidant and acidulant.

## Graphic abstract

**Figure:**
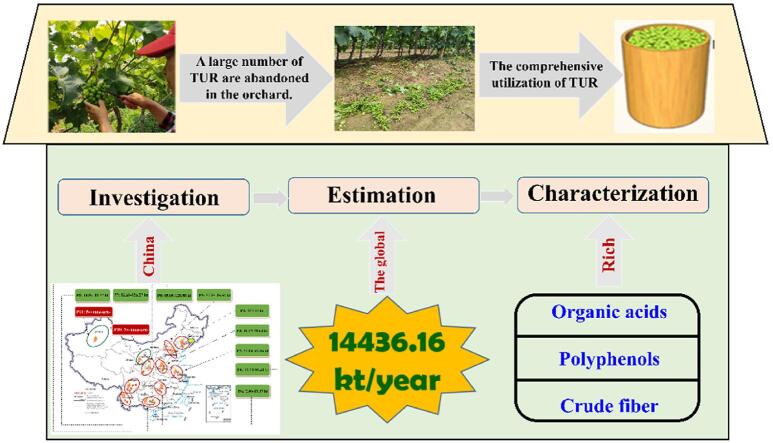

## 1 Introduction

According to the latest OIV (International Organisation of Vine and Wine) data, the world grape-planting area has been largely stable since 2016. In 2019, the global grape-planting area reached 7.4 Mha, including table grapes, wine grapes and dried varieties (OIV, 2019). To improve the quality of table grapes, fruit thinning is an indispensable cultivation and management step in the process of growing and developing fresh grapes. In the process of fruit thinning, if part of the developing young fruit is removed, the remaining young fruit can use more photosynthates, resulting in larger fruit grains and a more uniform fruit quality (Gutiérrez-Gamboa, Wei, Moreno-Simunovic, Sun, & Fang, 2020).

In addition, fruit thinning is also one of the most effective measures for increasing the return of flowers in the following year (Inglese, Barbera, La Mantia, & Portolano, 1995), reducing the effects of continuous cropping and balancing the ratio of fruit to buds (Costa, & Vizzotto, 2000). Besides table grapes, previous studies have also shown that wine grape varieties may increase in their contents of total anthocyanins and total polyphenols after fruit thinning (Gatti, Bernizzoni, Civardi, & Poni, 2012). With sustainable development and the evolution of the times, our goal is not only to produce high-quality grapes and wines, but also to pursue clean production of the entire industry chain. Therefore, the harmless, efficient and high-efficiency solid waste Value processing is imminent. The main solid wastes in the grape industry including grape pomace and wine lees (Muhlack et al., 2017), winter pruning branches (Manzone et al., 2016, Pari et al., 2017) have been systematically studied. In addition, the thinning of unripe fruits of other horticultural crops has also been extensively studied, such as apple (Chen et al., 2015, Chen et al., 2017, Choi et al., 2010, Dou et al., 2015, Sun et al., 2016), banana (Ritthiruangdej et al., 2011, Rosado et al., 2020, Segundo et al., 2017, Viana et al., 2018, Wang et al., 2012) and so on.

Due to the rapid expansion of table grape planting area and the continuous improvement of viticulture management, fruit thinning has become a very extensive horticultural measure, so a large amount of solid waste such as thinned unripe grapes (TUR) is generated every year (Guo et al., 2019, Fia et al., 2021, Wei et al., 2021). This new type of solid waste will be accumulated into the soil of the orchard and may become hosts for pathogens after decaying (Dou et al., 2015), which accelerates the spread of crop diseases and increases the acidity of the soil, thereby disrupting the microbial community in the soil (Mazzola, 1998). For example, Cesco et al. (2012) showed that a high concentration of phloridzin in apple roots could cause a replanting disease of apple trees, and Sun et al. (2017) also indicated that a high concentration of polyphenols might be associated with toxic behavior. Therefore, the abandonment of TUR is not only a great waste of agricultural resources but also places great pressure on the environment. If TUR resources are recyclable, the number of TUR abandoned in orchards will be greatly reduced and the negative impact of TUR on the soil will be addressed.

Therefore, TUR gradually attracts people's attention (Bovo et al., 2016, Fia et al., 2021, Gutiérrez-Gamboa et al., 2020, Piccardo et al., 2019, Tinello and Lante, 2017) and becomes an urgent problem for the grape industry. Previous studies have revealed that grape juice made from TUR could be used as food-flavoring agent (Dupas de Matos et al., 2018), such as replacing vinegar to make salad dressings and pickles. The results showed that the addition of TUR juice did not affect the original flavor; instead, its effect was to inhibit pathogenic bacteria such as *Salmonella Typhimurium*, *Bacillus cereus*, *Escherichia coli*, *Listeria monocytogenes* and *Staphylococcus aureus* (ŞenIz and Öncül, 2016, Turkmen et al., 2017). Moreover, TUR extract had an inhibitory effect on tyrosinase, polyphenol oxidase, etc., and could effectively inhibit enzymatic browning and oxidation (Honisch et al., 2019). Therefore, TUR has the potential to become a natural raw material for skin whitening in the cosmetics industry (Gutiérrez-Gamboa, Wei, Moreno-Simunovic, Sun, & Fang, 2020). Additionally, TUR extracts have significant effects in improving serum cholesterol levels (Zibaeenezhad et al., 2012), reducing blood sugar (Gutiérrez-Gamboa, Wei, Moreno-Simunovic, Sun, & Fang, 2020) and inhibiting cancer cell viability (Nasser et al., 2020). In conclusion, TUR abandoned in orchards has great application potential in the food and pharmaceutical industries in the future.

Therefore, as a potential agricultural resource, the comprehensive utilization of TUR is of great significance for ecology and the economy. Nutritional and functional components analysis of TUR can provide a theoretical basis and technical support for its comprehensive utilization, and thus represents the beginning of all work to follow. However, few studies have analyzed and compared the nutritional and functional components of TUR and ripe grape fruit (RGF). Therefore, this study investigated the biomass of TUR in major grape-growing regions in China, and then estimated the TUR resources generated annually in China and worldwide. In addition, nine table grape varieties with a wide cultivation area were selected for a comprehensive comparative analysis of the basic physicochemical, nutritional and functional components and antioxidant activities of TUR and RGF. The research results provide a theoretical basis for follow-up research and high-value development and utilization of TUR.

## 2 Materials and methods

### 2.1 Investigation and estimation of TUR biomass

In this study, the biomass of TUR was estimated at 22 survey sites in 11 provinces of China, covering most table grape-growing regions in China: Liaoning Province (P1), Hebei Province (P2), Jiangsu Province (P3), Henan Province (P4), Shaanxi Province (P5), Hunan Province (P6), Sichuan Province (P7), Yunnan Province (P8), Guangxi Province (P9), the Ningxia Hui Autonomous Region (P10) and Xinjiang Uygur Autonomous Region (P11), using the main cultivars in each region for investigation and estimation (Fig. S1). The survey was conducted during the fruit-thinning period of table grapes (from May 2020 to July 2021). Fifty trees were randomly selected from each survey site, and the TUR abandoned by farmers during fruit thinning was collected and weighed. Taking the average weight (kg/tree FW) fruit-thinning biomass per plant at the survey site, annual TUR biomass was calculated using the following equation (Sun et al., 2020):

BTUR=Btree×ρ×A

where B_tree_ is the TUR biomass of an individual tree (kg/tree FW) as determined in this study, ρ is the planting density (trees/ha) and A is the cultivated area (Mha).

According to the results of previous a TUR survey and the grape planting areas of various provinces in the China Agriculture Yearbook 2016 (the Ministry of Agriculture of the Republic of China, 2016), the TUR biomass was calculated for the grape-planting areas of all provinces and cities in China.

When using the Web of Science platform’s advanced search, at first, the search formula (TS=“unripe grape” OR TS=“young grape” OR TS=“cluster thinning grape” OR TS=“thinned grape” OR TS=“grape thinning” OR TS=“immature grape” OR TS=“remaining well-ripened grape”) was entered to retrieve relevant research literature related to fruit thinning, including titles, abstracts and keywords published during 1982–2021. Then, text-mining software tools such as Excel and Vosviewer were used to analyze the country/region and keywords of the literature in the search results. Furthermore, the countries and research hotspots of fruit thinning in grape growth were speculated.

### 2.2 Sampling and sample preparation

The plant samples used for nutritional and functional analysis were nine commercial table grape varieties widely planted: kyoho (KH), zaomi (ZM), shine Muscat (SM), summer black (SB), hutai 8 (HT), wagamichi (WI), jasmine grape (JG), zaoxiawuhe (ZX) and red globe (RG) (Fig. S2). Since the grape varieties, terroir and management techniques of the vineyards were different, the sampling times were determined according to the growth and development of table grapes. The samples of different varieties needed to be in the same phenological period. The TUR and RGF were collected in the thinning period and mature period, respectively (Coombe, 1995) (Table S1).

Once collected, 2 kg samples were randomly weighed, and the total soluble solids (TSS), total sugar content (TSC), pH and titratable acid (TA) were determined immediately after the whole fruit was pulped with a homogenizer (H-AE-DNBI1, Hurom, South Korea). When measuring other components, the homogenate was prepared instantly before determination. All indicators’ determination was carried out uniformly after sample collection.

### 2.3 Physicochemical characteristics

One hundred grapes were detached, counted and weighed to obtain their average berry mass (BM). The TSC and TA were determined by the OIV (2017) method OIV (International Organisation of Vine and Wine) (2017). The TSS was determined with a hand-held digital Atago PAL-1 m (Atago Co. Ltd., Japan) and indicated by the Brix°. The pH was measured with a Mettler Toledo FE20 Desktop pH Meter (Mettler Toledo Instruments Co., Ltd., Shanghai, China).

The determination of the organic acids in grape fruits by HPLC was modified based on Gao et al. (2004). HPLC detection (high-performance liquid chromatography, Waters 2699, USA) was conducted using a C18 chromatographic column (4.6 × 250 mm, 5 μm; CAPCELL PAK C18; Shiseido, Japan). We utilized mobile-phase (A) 0.02 M dipotassium phosphate (pH adjusted to 2.3 using phosphoric acid) and mobile-phase (B) methyl alcohol. Mobile-phase A:B = 99:1 was used for isometric elution at a flow rate of 0.5 mL/min. The retention time was used to identify the components of organic acids, and the concentration of organic acids in samples was calculated by external standard method (Destandau et al., 2005). All 11 standard materials (oxalic acid, tartaric acid, quinic acid, malic acid, shikimic acid, lactic acid, acetic acid, citric acid, fumaric acid, succinic acid and propionic acid) were purchased from Sigma-Aldrich (St. Louis, MO, USA).

### 2.4 Nutritional and functional indicators

The method of Zhang et al. (2020) was used to determine the mineral element content and total protein (TP). The determination of crude fiber (CF) was carried out according to the Chinese national standard GB/T 5009.10-2003 (The National Standard of China, 2003).

The total polyphenol (TPC), flavonoid (TFC), tannin (TTC) and flavan-3-ols (TFO) contents were measured by the Folin**-**Ciocalteu colorimetric method, aluminum chloride colorimetric assay, methylcellulose precipitation tannin assay and *p*-(dimethylamino)cinnamaldehyde (*p*-DMACA)-HCl assay, respectively. The results are expressed as milligrams of gallic acid equivalents (GAE) per gram (mg GAE/g), milligrams of rutin equivalents per gram (mg RTE/g), milligrams of catechin equivalents per gram (mg CTE/g) and milligrams of catechin equivalents per gram (mg CTE/g), respectively. The experiments were operated according to the work of Cheng et al. (2020).

### 2.5 Analysis of antioxidant capacity

Total antioxidant capacity (*T*-AOC) was determined by a *T*-AOC kit (Solarbio Science & Technology Co., Ltd., Beijing, China). Both the 2,2-Diphenyl-1-picrylhydrazyl (DPPH) and 2,2′-Azinobis-(3-ethylbenzthiazoline-6-sulphonate (ABTS) radical scavenging capacity and copper ion-reducing capacity (CUPRAC) were determined according to Cheng et al. (2020), and the results are expressed in millimoles of trolox equivalent antioxidant capacity (TEAC) per 100 g.

### 2.6 Statistical analysis

The results are expressed as the mean ± standard deviation of three measurements. SPSS 20 (IBM, USA) was used for one-way analysis of variance (ANOVA), and Duncan’s multivariate test (*p* < 0.05, *p* < 0.01). GraphPad Prism 8 (GraphPad Software, USA), Origin 9.1 (OriginLab, USA), Excel and Vosviewer were used for image rendering and data analysis.

## 3 Results and discussion

### 3.1 Investigation of TUR biomass from survey site

The survey results for the TUR biomass at each survey site are shown in Table S2. The survey found that the TUR biomass depended on many factors such as the grape load-management measures, fruit-thinning time and vineyard cultivation and management practices; therefore, the TUR biomass varied greatly between the investigated points. The TUR biomass (3.16 kg/tree FW) per plant in the P9-2 area was the largest, but the total TUR biomass was small due to the low planting density of vineyards. In contrast, the TUR biomass (1.33 kg/tree FW) in the P1 area was much higher than those in other areas. By referring to the 2016 Yearbook (Ministry of Agriculture of the Republic of China, 2016), it was inferred that the P1 area could reach 257.75 kt per year, making it the area with the largest total TUR biomass among all the survey points. P1 has the most fertile soil in China, which is located in one of the four famous black soil belts in the world and belongs to the warm temperate continental monsoon climate. The soil is mostly sandy brown soil, with organic matter content up to 5%, while the soil organic matter content in northwest China is only about 0.14% (Yang, 2019). The soil with high fertility efficiency can effectively improve grape yield, so it may also be one of the reasons for the highest TUR biomass. In addition, the TUR biomasses of the P6 and P8 areas were the lowest, with 0.12–0.2 and 0.13 kg/tree FW, respectively. Firstly, it can be seen from Table S2 that P6 and P8 had earlier fruit thinning and lower unripe fruit weight. In addition, the amount of bud in the previous winter pruning was probably less in these two areas, and the amount of fruit in the second year was controlled, so the fruit thinning biomass per plant was the lowest among all survey sites.

In general, the planting process of table grape is carried out with thinning flowers and fruits. There are generally 300 to 1500 flowers on a grape inflorescence. The thinning of grapes is generally achieved by pinching the tip of the inflorescence and shaping the inflorescence, not to thin out a single flower, but to thin out all levels of cob branches (ie spikelets) in the inflorescence. Pinching the tip also reduces the chance of soft tip or jug disease at the tip of the flowers. Fruit thinning is to make the cluster more tidy and the fruit larger. From the survey results, it could be concluded that the TUR biomass was the largest when resulting from thinning of clusters/fruits, while thinning flowers/fruits created the smallest biomass. Fundamentally, the time of grape load management was the main factor determining the TUR biomass. For example, the fruit-thinning time in the P1 area was the latest, and the corresponding TUR biomass was significantly higher than those in other regions (*p* < 0.05). There was no significant difference in TUR biomass between different grape varieties at the same survey site (*p* > 0.05), as shown by the P6-1 and P7-2 areas. However, the TUR biomasses for the same variety significantly differed between areas, as can be seen from the survey results for SM at different survey points. First of all, the TUR biomasses were affected by artificial cultivation and management conditions. In addition, they are closely influenced by the genotype × environment (G × E) interaction as well. A significant G × E interaction results from the changes in the magnitude of the diff erences among genotypes in different environments or from changes in their relative ranking (crossover interaction) (Mohammadi et al., 2010).

It is also worth mentioning that the P10 and P11 areas are large table grape-growing areas in China, but the advantages of management measures in place at these locations for grape-load management have not been popularized due to the remoteness of the locations. To obtain a greater grape yield, farmers do not carry out load management of the grape crop. However, according to local researchers and technical trainers, improving the fruit quality will be the main goal of western Chinese grape cultivation management, and grape load management is the necessary road to achieving this goal, which is being actively popularized through information offered to farmers. Therefore, fruit thinning is becoming the trend of cultivation management in western China, and in the future, western China will be the main acquisition area for TUR.

### 3.2 Estimation of total TUR biomass in China and globally

To clearly understand the resources and scale of TUR, and thus better develop and utilize this potential agricultural resource, this study estimated the total amount of TUR biomass in the world based on the results of the TUR biomass survey in the major viticulture areas in China. During the survey, 22 survey sites in 11 provinces of China were included, covering almost all grape-growing regions in China. Each region has a unique terroir and management methods. Based on our team’s rich experience of grape planting and production in China, spanning 36 years, these 22 survey sites were chosen as the most representative vineyards to provide a picture of the local fruit-thinning situation. Hence, the survey results for Chinese TUR biomass obtained by the research institute were representative and could be used to estimate the total TUR biomass produced worldwide each year. Unfortunately, no other teams have been found to be investigating TUR biomass so far. As the first team to carry out this research, we were not able to conduct a comprehensive investigation on the fruit-thinning biomass of all grape-growing regions in the world. Therefore, the estimation obtained in this research may not be the most accurate result but it is of reference significance. In order to have a more comprehensive understanding of TUR biological resources. It is hoped that other researchers interested in the study to conduct fruit thinning surveys in their local areas to expand the scope of the survey and provide more accurate results. We will provide valuable basic data for the development and application of TUR with our joint efforts.

The estimated results for the TUR biomass generated in China are shown in Table S2. The average TUR biomass in China is 0.51 kg/tree (FW) per year, and the average planting density is about 3800 trees/ha. According to the research results and the OIV annual report (OIV, 2019), we estimate that the world produces about 14,436.16 kt TUR every year (Table S3), among which the top-three countries, in descending order, are Spain, China and France.

Based on the advanced search on the Web of Science platform, a total of 96 pieces of literature related to TUR were retrieved. Through statistical analysis of the countries and keywords in the literature, we determined that research related to TUR had been found from countries with large areas of viticulture, such as Turkey, Italy, China, France and Spain (Fig. S3a), and it could be inferred that fruit-thinning management has also been carried out in these regions during the grape-growing process. Therefore, this study has very important practical significance for the estimation of the global fruit-thinning biomass. In addition, a keyword co-occurrence map (Fig. S3b) drawn by Vosviewer text-mining software showed that the most prominent hotspots for TUR research include antioxidants, thermotolerance, anthocyanins and verjuice. This indicates that if the above hot research on TUR can be applied to practice, the solid agricultural resources of TUR will be transformed into treasure, reducing the negative impact on orchard environment and soil and increasing the sustainable development of grape industry. Based on the huge scale of TUR agricultural resources, the development and utilization of TUR will have great practical significance for sustainable development of agricultural resources and the ecological environment, and nutritional and functional components analysis of TUR will be the precursor work to its development and utilization.

### 3.3 Physicochemical characteristics

Table 1 shows physicochemical information on TUR and RGF. The weight of RGF is significantly higher than that of TUR (*p* < 0.05). The weight of RGF from 2.76 to 13.32, which RG and JG varieties were the largest and smallest. Meanwhile, for TUR, SB has the highest weight (3.09 g), and ZX and JG have the lowest TUR weights. Generally, ZX and JG were thinned about 15 days after flowering, while SB was thinned 50 days after flowering (Table S1). In order to be closer to the production and application of grape industry, the sampling time in this paper is determined according to the fruiting time of each grape planting area. Due to the vast territory of China, the cultivation and management measures of each grape planting area will be affected by planting objectives, planting technology, climate and other factors, so the fruit thinning time of each region cannot be unified. We can see that the weight of TUR was not only related to the characteristics of the variety but also closely related to the thinning time, a finding that was consistent with the biomass survey results for TUR found in this study.

**Table 1:** Physicochemical Parameters in TUR and RGF

Cultivar		BM (g)	TSS (°Brix)	TSC (g/L)	pH	TA (g/L)	Organic acids										
							Oxalic acid (mg/L)	Tartaric acid (g/L)	Quinic acid (g/L)	Malic acid (g/L)	Shikimic acid (mg/L)	Lactic acid (g/L)	Acetic acid (g/L)	Citric acid (mg/L)	Fumaric acid (mg/L)	Succinic acid (g/L)	Propionic acid (g/L)
TUR	KH	1.75 ± 0.02i	3.20n	26.04 ± 4.91f	2.95l	24.10 ± 0.39d	42.30 ± 0.11f	4.75 ± 0g	—	6.87 ± 0c	19.76 ± 0d	0.16 ± 0b	—	101.89 ± 1.98f	0.57 ± 0j	—	—
	ZM	0.75 ± 0.05k	3.90l	26.36 ± 2.49f	3.03i	20.00 ± 0.36f	57.96 ± 0c	5.41 ± 0d	—	4.43 ± 0h	48.41 ± 0.01a	0.12 ± 0c	—	111.00 ± 0.18d	0.78 ± 0f	—	—
	SM	1.75 ± 0.07i	3.10o	27.06 ± 3.78f	3.01j	22.26 ± 0.34e	—	4.18 ± 0h	—	6.62 ± 0d	6.69 ± 0j	0.11 ± 0d	—	149.78 ± 0b	2.19 ± 0a	—	—
	SB	3.09 ± 0.10g	3.10o	31.92 ± 0.04f	2.95l	18.08 ± 1.51g	43.59 ± 0.13e	5.02 ± 0.01e	—	7.88 ± 0b	10.06 ± 0.02f	—	—	107.69 ± 0.63e	0.92 ± 0e	—	—
	HT	1.48 ± 0j	4.00k	31.14 ± 2.47f	2.89m	26.43 ± 0.20c	22.96 ± 0.36g	4.80 ± 0.01f	—	6.53 ± 0.02e	8.82 ± 0.11g	0.22 ± 0a	—	74.09 ± 0.85g	0.72 ± 0.01g	—	—
	WI	0.62 ± 0.01l	2.90p	33.76 ± 10.82f	2.86n	24.09 ± 0.48d	133.23 ± 0b	9.64 ± 0.02b	—	5.82 ± 0f	7.31 ± 0.01i	0.10 ± 0e	—	224.54 ± 0.13a	1.60 ± 0.02b	—	—
	JG	0.57 ± 0.03m	4.00k	25.47 ± 4.16f	3.04i	27.63 ± 0.29b	—	7.20 ± 0c	—	5.69 ± 0g	41.6 ± 0.02b	—	—	69.34 ± 0.25h	1.50 ± 0c	—	—
	ZX	0.49 ± 0.02n	4.50j	31.75 ± 7.31f	2.94l	29.57 ± 0.55a	159.53 ± 0.98a	10.88 ± 0.02a	—	9.75 ± 0.01a	35.17 ± 0.09c	—	—	122.12 ± 0.84c	—	—	—
	RG	2.76 ± 0.20h	3.30m	27.20 ± 3.80f	2.97k	22.63 ± 0.14e	47.32 ± 0d	3.75 ± 0.06i	—	6.82 ± 0c	2.31 ± 0.01p	—	—	37.81 ± 3.39k	0.95 ± 0.01d	—	—
RGF	KH	4.36 ± 0.20f	18.30a	166.67 ± 4.93a	3.96e	6.45 ± 0.20i	—	0.92 ± 0n	—	1.33 ± 0.01k	4.84 ± 0k	0.09 ± 0f	—	28.00 ± 0.19p	0.28 ± 0o	—	—
	ZM	7.06 ± 0.17e	14.00d	145.51 ± 8.46b	4.02d	3.68 ± 0.30kl	1.94 ± 2.36i	1.05 ± 0.01m	—	0.84 ± 0.01p	10.57 ± 0.14e	0.08 ± 0g	—	29.82 ± 0.42n	0.32 ± 0n	—	—
	SM	6.79 ± 0.18e	11.30i	133.07 ± 12.10c	4.20a	3.10 ± 0.01 l	—	0.81 ± 0p	—	1.28 ± 0l	2.23 ± 0q	0.08 ± 0g	—	37.58 ± 0l	0.60 ± 0i	—	—
	SB	9.11 ± 0.18d	12.00g	121.69 ± 0d	3.70h	5.67 ± 0.13i	—	0.98 ± 0n	—	1.53 ± 0j	2.90 ± 0m	—	—	29.16 ± 0o	0.35 ± 0m	—	—
	HT	12.26 ± 0.38b	11.70h	94.87 ± 4.79e	3.72g	7.72 ± 0.22h	—	0.93 ± 0.01o	—	1.26 ± 0.01m	2.65 ± 0n	0.10 ± 0e		22.44 ± 0.14q	0.31 ± 0n	—	—
	WI	10.21 ± 0.20c	16.30b	163.33 ± 3.73a	4.05c	4.67 ± 0.09j	16.99 ± 0.54h	1.90 ± 0.02k	—	1.12 ± 0.04n	2.35 ± 0.16o	0.08 ± 0g	—	52.53 ± 0.07i	0.48 ± 0k	—	0.03 ± 0
	JG	2.76 ± 0.15h	14.80c	134.51 ± 3.74c	3.73g	7.30 ± 0.43h	—	1.41 ± 0.01l	—	1.09 ± 0.01o	9.21 ± 0.03f	—	—	21.49 ± 0.08r	0.46 ± 0l	—	—
	ZX	6.90 ± 0.06e	12.60f	151.50 ± 2.50b	3.89f	6.42 ± 0.03i	22.25 ± 1.18g	2.15 ± 0.02j	—	1.91 ± 0.01i	7.92 ± 0.21h	—	—	32.04 ± 0.63m	—	—	—
	RG	13.32 ± 0.22a	13.50e	122.16 ± 1.98d	4.08b	3.92 ± 0.14jk	—	0.72 ± 0.02q	—	1.32 ± 0.04k	3.14 ± 0.06l	—	—	45.30 ± 1.48j	0.64 ± 0.01h	—	—

Note: Data among nine cultivars were analyzed through one-way ANOVA and different letters in the column indicate significant differences among nine cultivars at the 0.05 level.

Abbreviations: KH, Kyoho; ZM, Zaomi; SM, Shine muscat; SB, Summer black; HT, Hutai; WI, Wagamichi; JG, Jasmine grape; ZX, Zaoxiawuhe; and RG, Red globe.

During grape growth, the permeability of the cell membrane gradually increases so that the acid stored in the cell vacuole is respirated and converted from acid to sugar (Jediyi et al., 2019). As shown in Table 1, the TSS and TSC of TUR are generally lower, ranging from 2.9 to 4.5°Brix and 25.47–33.76 g/L, respectively. The TSS and TSC of the same variety in RGF are significantly higher than those of TUR (*p* < 0.05). Their TSS and TSC are 11.3–18.3°Brix and 94.87–166.67 g/L, respectively. In addition, the TA content of TUR is significantly higher than that of RGF, their contents ranged from 18.08 to 29.57 g/L and 3.10 to 7.72 g/L, respectively, while the pH is significantly lower than that of RGF (p < 0.05). Among them, the TA of ZX in TUR is the highest, this may be due to the fact that ZX is an extremely early maturing variety, and its fruit thinning time and maturity time are earlier than other varieties. When it is at maturity, the TA content is reduced to 6.42 g/L, which is about one third of the TA content in TUR. Therefore, ZX in TUR represents a good natural acidifier and a natural raw material with great potential in the functional food industry.

Table 1 shows that the contents of tartaric acid and malic acid in TUR are 3.75–10.88 g/L and 4.43–9.75 g/L, respectively; the contents of tartaric acid and malic acid in RGF are 0.72–2.15 g/L and 0.84–1.53 g/L, respectively. However, malic acid and tartaric acid were the most abundant organic acids in both fruiting and ripening stages of grapes (Table 1). Previous studies have shown that tartaric acid and malic acid account for 69–92% of grape fruit organic acids (Angeli et al., 2013, Conde et al., 2007). Due to glycolysis, the Krebs cycle, the glyoxylic acid cycle and shikimic acid pathways, other organic acids were also produced in grape fruits (Fuleki et al., 1993, Liu et al., 2006). As shown in Table 1, citric acid and shikimic acid were detected in all grape varieties, among which WI and ZM had the highest contents. Meanwhile, quinic acid, acetic acid, succinic acid and propionic acid were not detected among all RGF and TUR.

### 3.4 Nutrition and function

#### 3.4.1 Mineral elements

The contents of eight mineral elements in TUR and RGF of different grape varieties are shown in Table 2. The results show that the calcium contents of TUR and RGF are rich, at 3324.62–4309.16 mg/100 g, which is significantly higher than the ranges for other mineral elements (*p* < 0.05). Calcium is followed by K and Mg, with contents of 79.15–249.62 mg/100 g and 44.61–62.31 mg/100 g, respectively.

**Table 2:** Mineral Elements in TUR and RGF

Cultivar		Ca (mg/100g)	K (mg/100g)	Mg (mg/100g)	P (mg/100g)	Na (mg/100g)	Fe (mg/100g)	Cu (mg/100g)	Zn (mg/100g)
TUR	KH	3643.64 ± 273.9abc	102.81 ± 6.17h	46.08 ± 3.11cde	7.47 ± 0.90g	9.17 ± 0.80b	5.84 ± 1.99bcde	3.31 ± 0.20d	0.21 ± 0.09fg
	ZM	3984.15 ± 315.82abc	155.78 ± 9.26fg	52.24 ± 5.49abcde	13.47 ± 0.64de	10.08 ± 0.77ab	9.66 ± 6.10a	7.51 ± 0.03b	0.25 ± 0fg
	SM	3919.29 ± 78.68abc	96.76 ± 2.46h	50.96 ± 1.62bcde	8.94 ± 0.17g	10.28 ± 0.50ab	8.04 ± 1.63ab	0.40 ± 0.23fg	0.35 ± 0.08def
	SB	4286.08 ± 438.26a	79.15 ± 0.93h	55.51 ± 8.20abc	7.86 ± 0.27g	10.63 ± 1.31ab	3.22 ± 0.32e	10.39 ± 0.98a	0.27 ± 0.08efg
	HT	4159.68 ± 691.45ab	95.47 ± 8.62h	52.88 ± 6.70abcde	7.38 ± 0.41g	10.85 ± 2.28ab	3.39 ± 0.50de	10.32 ± 0.72a	0.78 ± 0.13b
	WI	4219.08 ± 358.17ab	175.48 ± 33.30ef	62.31 ± 12.33a	21.84 ± 2.59b	11.16 ± 1.50ab	6.96 ± 0.94abcde	5.24 ± 0.60c	0.36 ± 0.10def
	JG	4210.72 ± 107.35ab	131.78 ± 8.02g	59.60 ± 0.52ab	20.41 ± 0.17b	10.64 ± 0.39ab	7.27 ± 0.97abc	3.54 ± 0.42d	0.35 ± 0.13def
	ZX	4109.87 ± 714.36abc	215.35 ± 9.66cd	58.73 ± 7.57ab	26.74 ± 0.86a	11.20 ± 1.69ab	5.87 ± 1.16bcde	3.86 ± 0.16d	0.66 ± 0.21bcd
	RG	3950.07 ± 546.24abc	104.48 ± 6.21h	51.24 ± 4.01bcde	11.49 ± 0.09f	13.01 ± 4.80a	6.42 ± 0.77abcde	10.80 ± 0.94a	0.59 ± 0.04bcde
RGF	KH	3892.30 ± 707.11abc	234.63 ± 9.95abc	49.35 ± 7.70bcde	17.77 ± 3.23c	11.82 ± 2.61ab	5.67 ± 1.10bcde	2.05 ± 0.80e	0.41 ± 0.24cdef
	ZM	4036.64 ± 663.77abc	194.68 ± 0.01de	56.26 ± 5.37abc	18.20 ± 0.84c	11.30 ± 1.80ab	5.29 ± 0.71bcde	0.20 ± 0fg	0.36 ± 0.17def
	SM	3426.01 ± 379.56bc	178.74 ± 7.70ef	44.11 ± 4.46e	14.55 ± 0.48de	9.62 ± 1.24b	6.17 ± 1.90abcde	—	—
	SB	4009.03 ± 80.37abc	249.62 ± 8.75g	51.37 ± 4.10bcde	8.69 ± 0.17g	10.70 ± 0.10b	5.95 ± 1.36 abcde	—	0.14 ± 0.12fg
	HT	4309.16 ± 164.73a	184.12 ± 2.15e	54.14 ± 0.56abcde	12.94 ± 0.24ef	11.20 ± 0.58ab	5.52 ± 0.40bcde	0.92 ± 0.04f	0.82 ± 0.13b
	WI	3324.62 ± 103.82c	241.74 ± 8.35ab	44.61 ± 1.79de	20.79 ± 0.65b	9.17 ± 0.21ab	4.11 ± 0.44cde	—	0.17 ± 0.07fg
	JG	3722.58 ± 413.38abc	225.15 ± 10.67abc	53.92 ± 5.68abcde	21.93 ± 0.10b	10.25 ± 0.39ab	7.86 ± 0.78abc	—	1.35 ± 0.90a
	ZX	3906.52 ± 203.04abc	218.12 ± 27.14bcd	55.25 ± 3.03abcd	21.76 ± 0.20b	10.67 ± 0.10ab	5.50 ± 2.19bcde	—	0.70 ± 0.34bc
	RG	3703.94 ± 257.23abc	249.62 ± 31.12a	51.04 ± 0.02bcde	15.24 ± 0.10d	11.25 ± 0.05ab	7.18 ± 2.71abcd	—	0.60 ± 0.28bcd

Note: Data among nine cultivars were analyzed through one-way ANOVA and different letters in the column indicate significant differences among nine cultivars at the 0.05 level.

Abbreviations: KH, Kyoho; ZM, Zaomi; SM, Shine muscat; SB, Summer black; HT, Hutai; WI, Wagamichi; JG, Jasmine grape; ZX, Zaoxiawuhe; and RG, Red globe

**Fig. 1:**
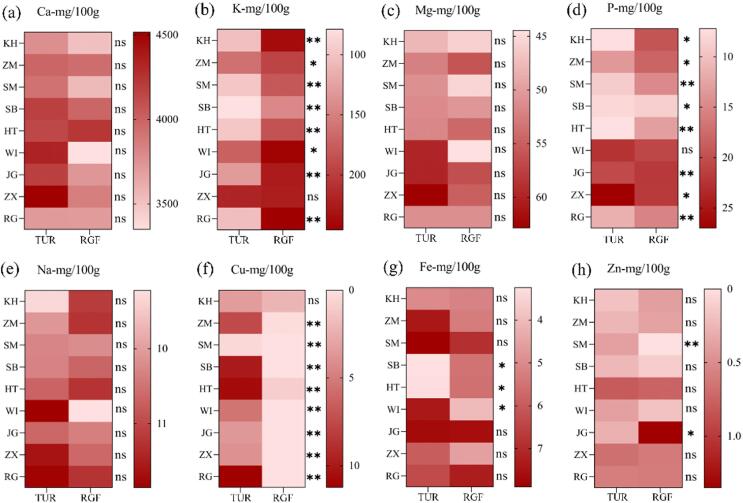
The mineral elements of TUR and RGF, (a) Ca, (b) K, (c) Mg, (d) P, (e) Na, (f) Cu, (g) Fe, (h) Zn.

Feige et al. (2014) also mentioned that K, Ca and Mg are the most abundant mineral elements in grape fruits. Mg and Ca participate in cell development and are components of cell walls. K and Mg are cofactors of various enzymes involved in carbohydrate metabolism, and they exist at higher concentrations during the growth phase. In addition, five mineral elements—P, Na, Fe, Cu and Zn—were detected in TUR and RGF, while Cu and Zn were detected in only some varieties of RGF. Fig. 1 shows the differences between TUR and RGF for eight mineral elements. Among them, zinc is required for multiple metabolic processes as a structural, regulatory, or catalytic ion (King et al., 2016). Only the RGF of SM did not detect the presence of zinc, and the zinc content of other samples ranged from 0.14 to 1.35 mg/100 g. Compared with RGF, the zinc content of TUR was more uniform. Except for ZX, the content of K in RGF of other table grape varieties is significantly higher than that of TUR (*p* < 0.05), and with the ripening of fruit, the Ca and Mg contents of most varieties decrease, with similar results also observed in the studies of Feige et al., 2014, Mahmood et al., 2012. The Cu content of all TUR varieties was higher than that of RGF, and all varieties demonstrated significance except for KH (*p* < 0.01). For other mineral elements, TUR and RGF do not have obvious rules for different varieties. In addition to varieties’ characteristics, climatic conditions and the soil pH are also major factors affecting the mineral element content (Zhang et al., 2020).

#### 3.4.2 CF and TP

**Fig. 2:**
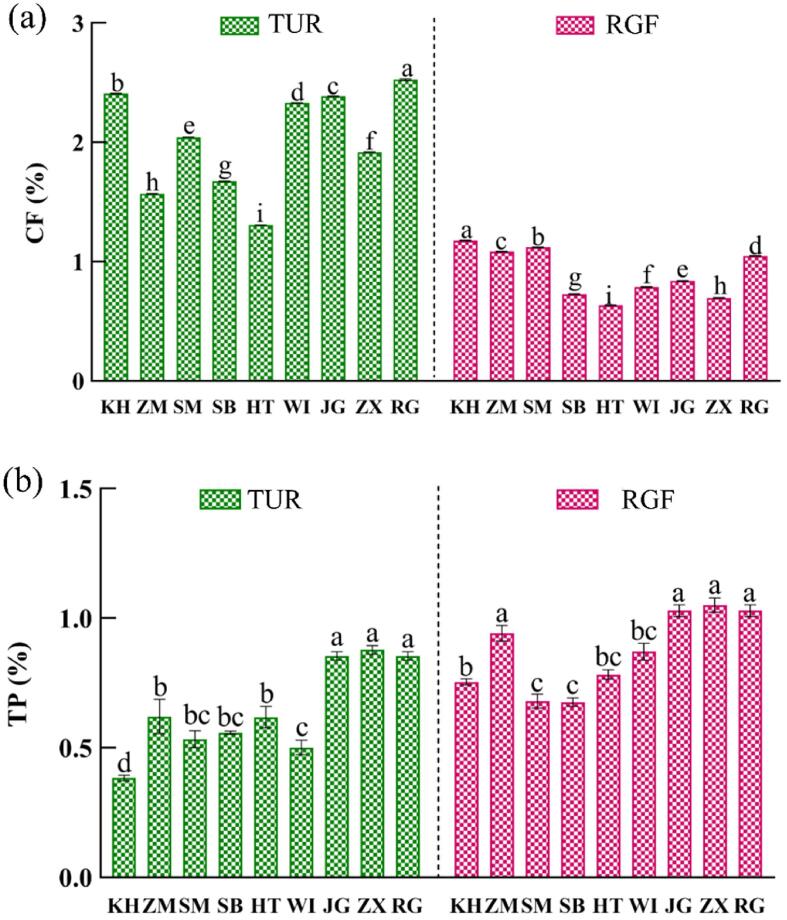
The content of crude fiber and total protein in TUR and RGF, (a) crude fiber (CF), (b) total protein (TP).

Studies have shown that a higher CF content in food can improve bodily health (Honeck et al., 2020), such as by reducing the risk of gastric ulcers (Di Martino et al., 2013), reducing toxin production (Wenk, 2001), preventing constipation, etc. (da Silva, van den Borne, Gerrits, Kemp, & Bolhuis, 2012). The CF of nine TUR and RGF varieties is shown in Fig. 2a, with the crude fiber content of TUR 1.4–3.0 times that of RGF, and the CF of different TUR varieties at 1.30–2.53%, among which RG has the highest CF in TUR, while HT has the lowest. The TP of nine TUR and RGF varieties is shown in Fig. 2b, with the TP content of RGF 1.2–1.9 times that of TUR. The TP content of each variety is 0.39–0.87% in TUR and 0.67–1.04% in RGF. The TP content increases gradually during grape ripening, which is consistent with the findings of previous studies (Riu-Aumatell et al., 2002, Zhu et al., 2017). And simultaneously, the protein of TUR and RGF present major differences in composition and structure (Deytieux et al., 2007). The characterization of proteins is apparently an essential parameter for understanding grape ripening. For example, as the berry ripens, it a general decrease of glycolysis, an increase of PR proteins in the range of 2035 kDa (Marzia, Iolanda, François-Xavier, & Andrea, 2007) and increases in the abundance of different chitinase and b-1,3-glucanase isoforms. At the beginning of colour-change, proteins involved in photosynthesis, carbohydrate metabolisms, and stress response are identified as being over-expressed (Deytieux et al., 2007).

#### 3.4.3 TPC, TFC, TTC and TFO

**Fig. 3:**
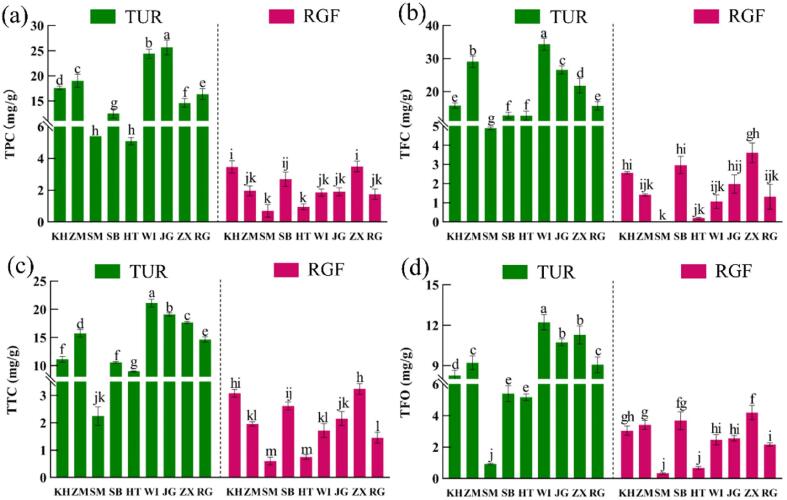
The content of phenolic components in TUR and RGF (a) total phenols (TPC), (b) total flavonoids (TFC), (c) total tannins (TTC), and (d) total flavan-3-ols (TFO).

Polyphenols can protect the nervous system, reduce inflammation, reduce the incidence of diabetes and cardiovascular disease and inhibit the occurrence of tumors (Di Lorenzo et al., 2019). There are two peaks of TFC in the whole grape fruit development process. The first peak appears about 20 days after flowering (Colombo et al., 2019), just in the fruit-thinning period of table grapes. From Fig. 3a–d, it can be seen that the phenolic substances in nine TUR were extremely rich, and the contents of TPC, TTC, TFC and TFO were significantly higher than those of RGF (*p* < 0.05). Specifically, the TPC, TFC, TTC and TFO of TUR were 4.2–13.5 (Fig. 3a), 3.6–12.3 (Fig. 3b), 4.3–62.8 (Fig. 3c) and 1.5–7.6 times (Fig. 3d) those of RGF, respectively. Furthermore, it was found that among the TUR, JG had the highest TPC (25.65 mg/g) and WI had the highest TTC, TFC and TFO contents, so the TUR of JG and WI were rich sources of phenolics.

### 3.5 Antioxidant activities

**Fig. 4:**
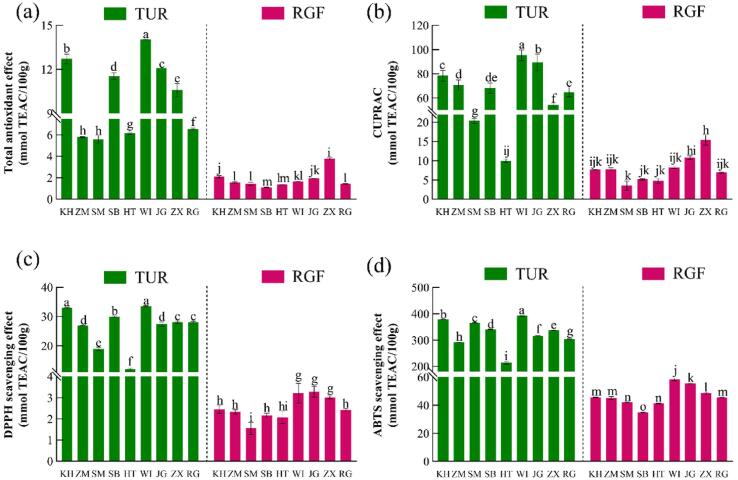
The antioxidant capacity of TUR and RGF (a) total antioxidant capacity, (b) copper ion reducing capacity, (c) DPPH radical scavenging capacity, and (d) ABTS radical scavenging capacity.

It is well-known that grapes are a fruit with a high antioxidant capacity (Colombo et al., 2019). However, as shown in Fig. 4, TUR was significantly higher than RGF (*p* < 0.05), which showed there was an extremely high antioxidant capacity. The *T*-AOC (Fig. 4a), CUPRAC (Fig. 4b), DPPH and ABTS radical scavenging capacities (Fig. 4c, d) were 2.8–10.3, 5.8–13.9, 5.2–9.7 and 2.1–13.1 times those of RGF, respectively. According to relevant literature, the antioxidant activity of most TUR varieties (such as WI, KH, JG, ZX, SB, etc.) is much higher than that of kiwifruit (Zhang et al., 2020), apple (Li et al., 2020), blueberry (Castrejón, Eichholz, Rohn, Kroh, & Huyskens-Keil, 2008), strawberry (Scalzo, Politi, Pellegrini, Mezzetti, & Battino, 2005) and other fruits with high antioxidant capacities. Therefore, TUR has the potential to become a natural antioxidant, which greatly improves its added value and constitutes the direction of further utilization of TUR. This can reduce the soil and environmental pressure brought by long-term accumulation of TUR in orchards, and will promote increased income and quality in the grape industry.

## 4 Conclusions

As one of the most widely distributed fruits in the world, grapes have a large number of TUR abandoned in orchards during the fruit-thinning stage of the harvest. According to the investigation and estimation of the biomass of TUR, about 1695.75 kt TUR is produced in China every year, and as much as 14436.16 kt worldwide. The fruit-thinning time is the important factor affecting the biomass of TUR, which also somewhat affects the nutrient content of TUR. In our research, compared with the RGF, the TP and TSC of each TUR variety were lower, but the grapes contained large amounts of TA (in particular, the levels of tartaric acid and malic acid were five times those of RGF) and CF. The polyphenols TPC, TFC, TTC and TFO of TUR were 4.2–13.5, 3.6–12.3, 4.3–62.8 and 1.5–7.6 times those of RGF, respectively, and their antioxidant capacity was significantly higher than that of RGF. In conclusion, TUR may be a potential natural antioxidant and acidifier due to its rich bioactive components such as organic acids, polyphenols and crude fibers. If TUR can be utilized, this will bring considerable economic and ecological benefits. How to strike a balance between the fruit-thinning time to ensure the quality of table grapes and the value of TUR remains to be clarified in subsequent studies. The results presented here provide a theoretical basis for follow-up research and high-value development and utilization of TUR.

## CRediT authorship contribution statement

**Mengyuan Wei:** Methodology, Investigation, Data curation, Visualization, Writing – original draft. **Tingting Ma:** Methodology, Investigation, Data curation, Visualization. **Muming Cao:** Investigation, Resources. **Binsheng Wei:** Investigation, Resources. **Chao Li:** Investigation, Resources. **Caihong Li:** Conceptualization, Writing – review & editing. **Kekun Zhang:** Conceptualization, Writing – review & editing. **Yulin Fang:** Conceptualization. **Xiangyu Sun:** Conceptualization, Supervision, Writing – review & editing.

## Declaration of Competing Interest

The authors declare that they have no known competing financial interests or personal relationships that could have appeared to influence the work reported in this paper.
