# Supplementary material for: Biomass estimation and characterization of the nutrient components of thinned unripe grapes in China and the global grape industries
Source: Food Chem X. 2022 Jun 16;15:100363. doi: 10.1016/j.fochx.2022.100363 (PMC9214794; doi:10.1016/j.fochx.2022.100363)
Supplement: Supplementary data 1 [file mmc1.docx]

**Figure S1. The investigation of TUR biomass in main grape cultivation regions from China (P1: Liaoning Province, P2: Hebei Province, P3: Jiangsu Province, P4: Henan Province, P5: Shaanxi Province, P6: Hunan Province, P7: Sichuan Province, P8: Yunnan Province, P9: Guangxi Zhuang Autonomous Region, P10: Ningxia Hui Autonomous Region, P11: Xinjiang Uygur Autonomous Region).**

**Note: the survey of P9 was conducted in 2020, and the survey of P1-P8, P10, P11 in 2021**


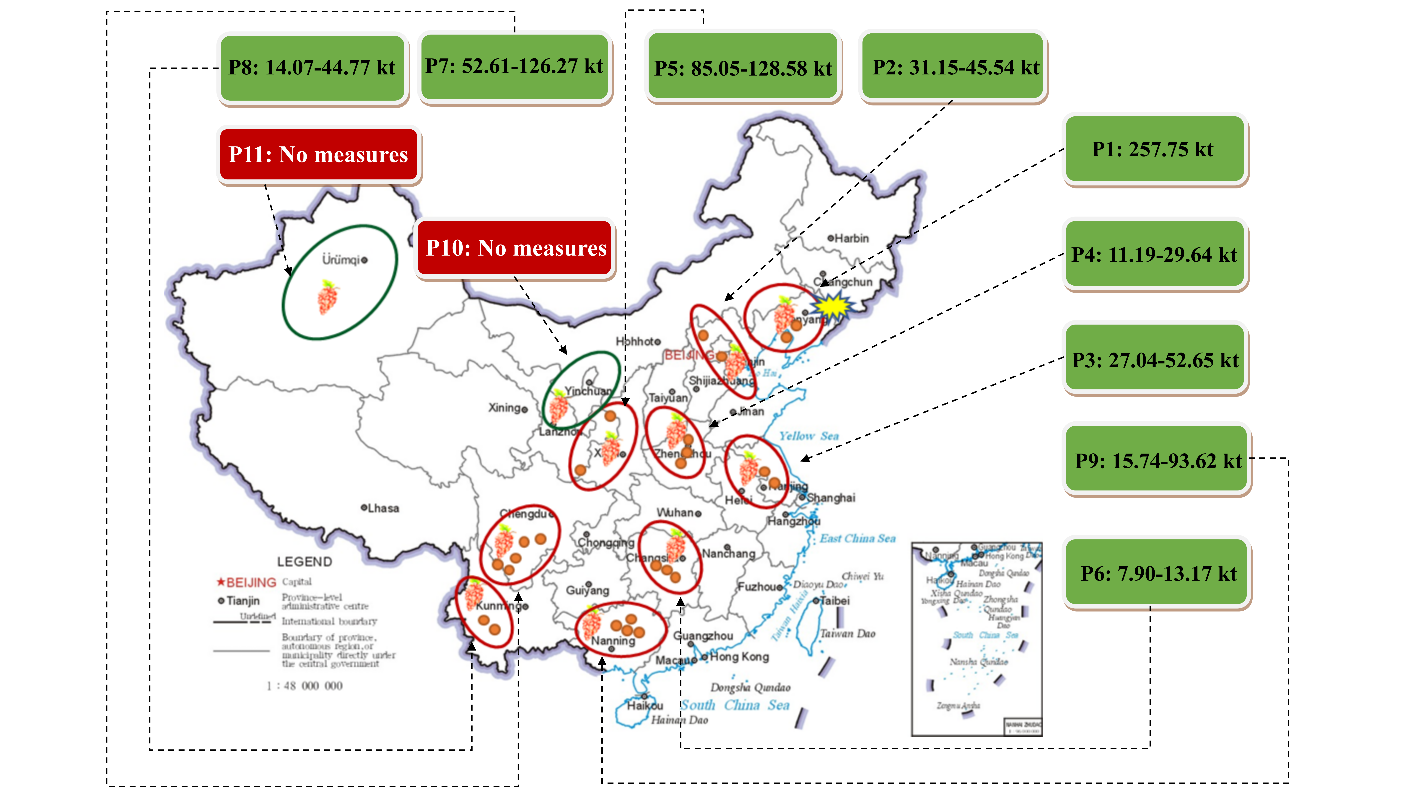


**Figure S2. The nine varieties of TUR and RGF**

**
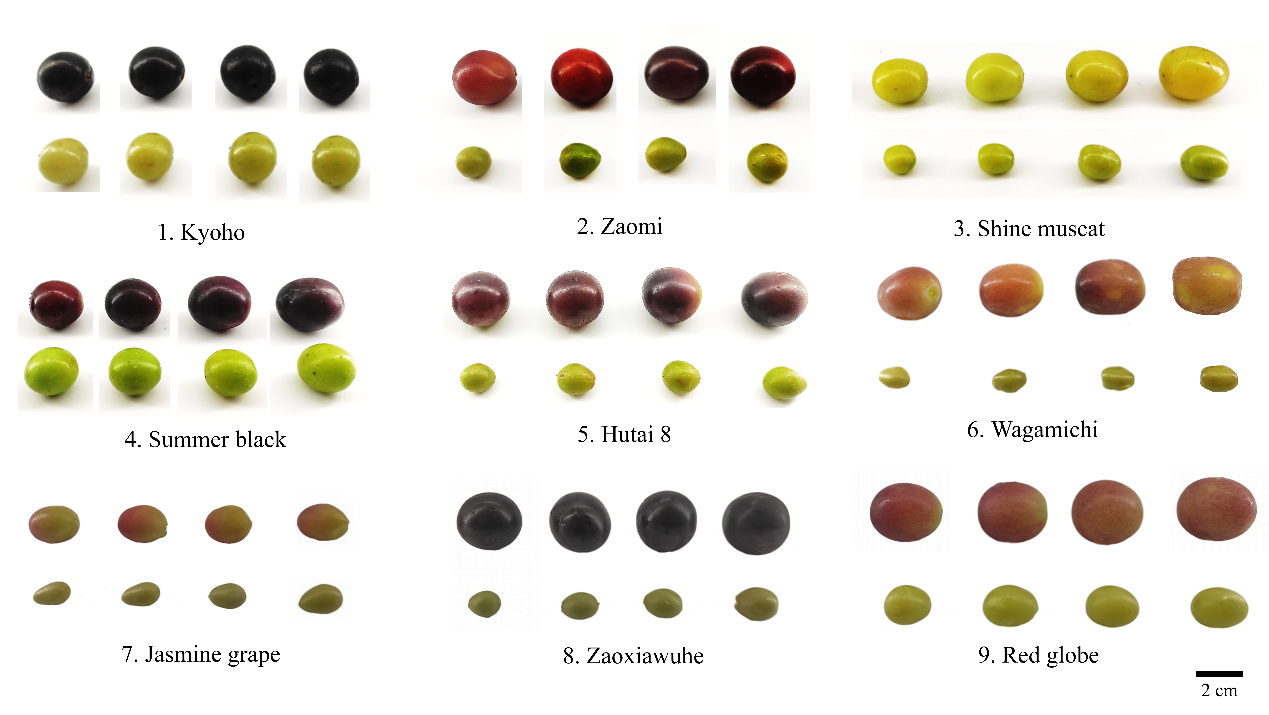
**

**Figure S3. (a) The number of TUR related articles published in each country and (b) keywords co-occurrence and research trend visualization.**

(a)

**
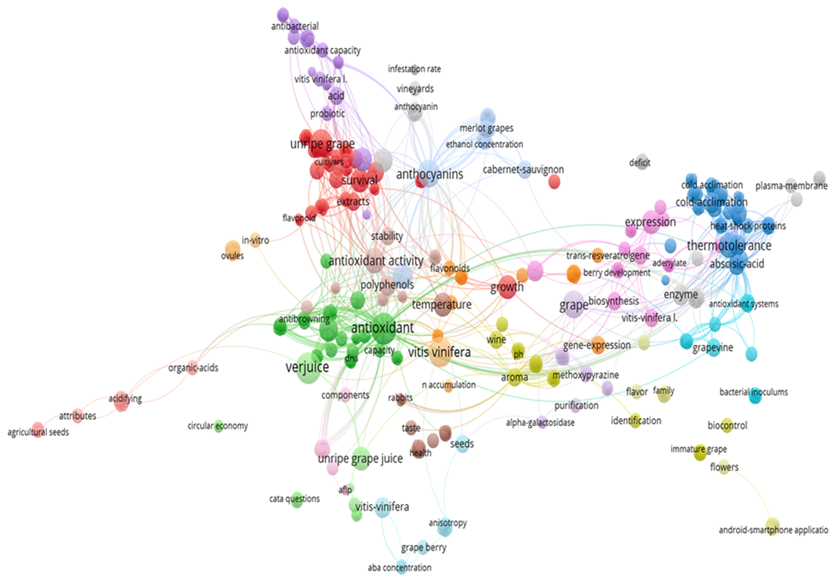

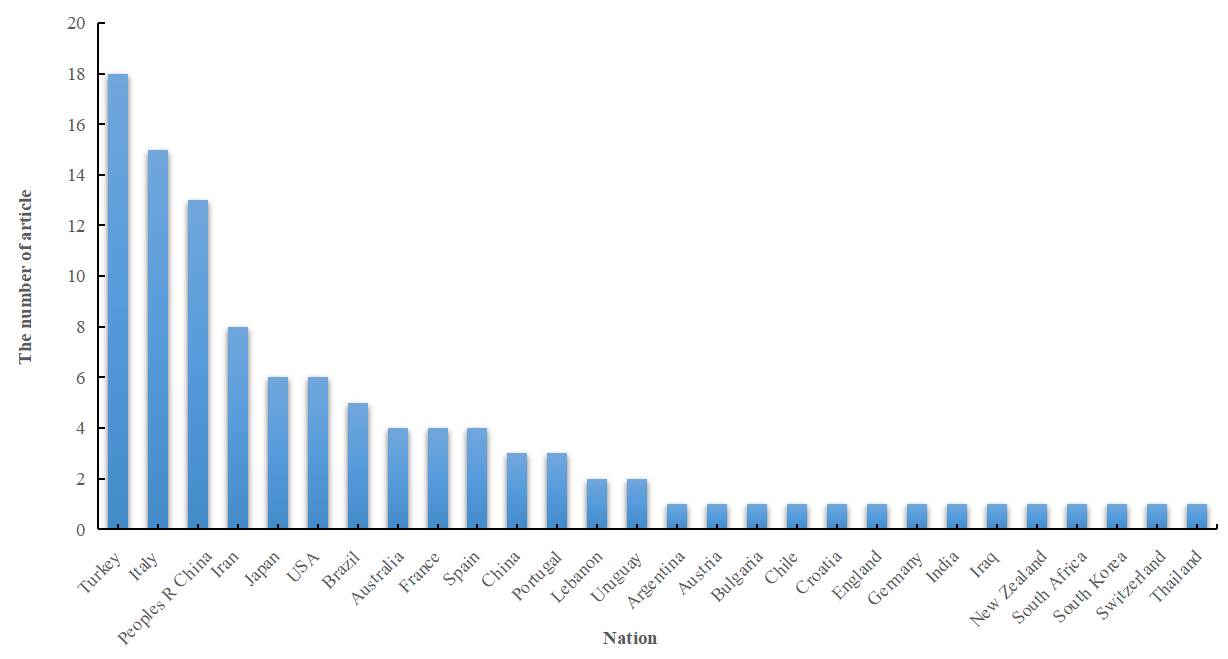
**

(b)

**Table S1. Cultivar, Thinning period, Harvest period and Region of grapes.**

| **Cultivar** | **Abbri.** | **Thinning period**  **（**after flowers**）** | **Phenological stages** ^a^ | **Harvest period**  **（**after flowers**）** | **Region** |
| --- | --- | --- | --- | --- | --- |
| Kyoho | KH | 15 days | E-L33 | 85 days | Guangxi,China |
| Zaomi | ZM | 45 days | E-L33 | 85 days | Shaanxi,China |
| Shine muscat | SM | 50 days | E-L33 | 80 days | Shaanxi,China |
| Summer black | SB | 50 days | E-L33 | 80 days | Shaanxi,China |
| Hutai 8 | HT | 20 days | E-L33 | 65 days | Shaanxi,China |
| Wagamichi | WI | 15 days | E-L33 | 65 days | Shaanxi,China |
| Jasmine grape | JG | 15 days | E-L33 | 65 days | Shaanxi,China |
| Zaoxiawuhe | ZX | 15 days | E-L33 | 65 days | Shaanxi,China |
| Red globe | RG | 30 days | E-L33 | 110 days | Shaanxi,China |

a: phenological stages refers E-L system (Coombe et al.,1995)

**Table S2 The investigation of TUR biomass in China.**

| Province | Area (kha) | Survey site | Cultivar | Measures of crop load management | Thinning fruits period  （after flowers） | B _tree_  (kg/tree FW) | | Planting density  (tree /ha) | B_TUR_ (kt) |
| --- | --- | --- | --- | --- | --- | --- | --- | --- | --- |
| P1 | 32.3 | P1 | KH | Thinning clusters/fruits | 60 days | 1.33 | 1.33 | 6000 | 257.75 |
| P2 | 43.9 | P2-1 | SB | Thinning fruits | 23 days | 0.52 | 0.37 | 1995 | 31.15-45.54 |
|  |  | P2-2 | MB | Thinning flowers/fruits | 38 days | 0.22 |  | 3225 |  |
| P3 | 34.8 | P3-1 | SB | Thinning flowers/fruits | 18 days | 0.21 | 0.31 | 3700 | 27.04-52.65 |
|  |  | P3-2 | SM |  | 20 days | 0.41 |  | 3900 |  |
| P4 | 42 | P4-1 | SB | Thinning flowers/fruits | 8 days | 0.536 | 0.51 | 840 | 11.19-29.64 |
|  |  | P4-2 | SM |  | 20 days | 0.84 |  | 840 |  |
|  |  | P4-3 | SM |  | 16 days | 0.16 |  | 1665 |  |
| P5 | 47 | P5-1 | RG | Thinning fruits | 30 days | 0.61 | 0.56 | 4485 | 85.05-128.58 |
|  |  | P5-2 | HT |  | 20 days | 0.52 |  | 3480 |  |
| P6 | 26.6 | P6-1 | SSG | Thinning flowers/fruits | 15 days | 0.2 | 0.15 | 2475 | 7.90-13.17 |
|  |  |  | YB |  | 15 days | 0.14 |  | 2475 |  |
|  |  |  | SM |  | 15 days | 0.12 |  | 2475 |  |
| P7 | 35.5 | P7-1 | CG | Thinning fruits | 7 days | 0.45 | 0.46 | 7410 | 52.61-126.27 |
|  |  | P7-2 | CG |  | 6 days | 0.5 |  | 3705 |  |
|  |  |  | SM |  | 5 days | 0.4 |  | 3705 |  |
|  |  |  | SSG |  | 15 days | 0.48 |  | 7410 |  |
|  |  | P7-3 | CG |  | 13 days | 0.48 |  | 4170 |  |
| P8 | 32.8 | P8-1 | RG | Thinning fruits | 18 days | 0.13 | 0.13 | 10500 | 14.07-44.77 |
|  |  | P8-2 | SM |  | 15 days | 0.13 |  | 3300 |  |
| P9 | 39.5 | P9-1 | SB | Thinning flowers/fruits | 22 days | 0.27 | 0.99 | 7800 | 15.74-93.62 |
|  |  | P9-2 | SM |  | 32 days | 3.16 |  | 750 |  |
|  |  | P9-3 | SM |  | 43 days | 0.21 |  | 3300 |  |
|  |  | P9-4 | SM |  | 15 days | 0.32 |  | 1245 |  |
| P10 | 27.2 | P10-1 | No measures of crop load management | | | | | | |
| P11 | 140.6 | P11-1 |  |  |  |  |  |  |  |
| National average | | | | | | 0.51 | | 3800 |  |

**Table S3 The estimation of TUR biomass in the world.**

| Nation | Area (kha) | B_TUR_ (kt FW) |
| --- | --- | --- |
| Spain | 969.00 | 1877.92 |
| China | 875.00 | 1695.75 |
| France | 793.00 | 1536.83 |
| Italy | 705.00 | 1366.29 |
| Turkey | 448.00 | 868.22 |
| USA | 439.00 | 850.78 |
| Argentina | 218.00 | 422.48 |
| Chile | 212.00 | 410.86 |
| Portugal | 192.00 | 372.10 |
| Romania | 191.00 | 370.16 |
| Iran | 153.00 | 296.51 |
| India | 151.00 | 292.64 |
| Moldova | 147.00 | 284.89 |
| Australia | 146.00 | 282.95 |
| South Africa | 126.00 | 244.19 |
| Uzbekistan | 111.00 | 215.112 |
| Greece | 106.00 | 205.43 |
| Germany | 103.00 | 199.61 |
| Afghanistan | 94.00 | 182.17 |
| Russia | 92.00 | 178.30 |
| Egypt | 84.00 | 162.79 |
| Brazil | 82.00 | 158.92 |
| Algeria | 75.00 | 145.35 |
| Hungary | 69.00 | 133.72 |
| Bulgaria | 66.00 | 127.91 |
| Georgia | 55.00 | 106.59 |
| Austria | 49.00 | 94.96 |
| Morroco | 46.00 | 89.15 |
| Syria | 45.00 | 87.21 |
| Ukraine | 42.00 | 81.40 |
| New-zealand | 39.00 | 75.58 |
| Mexico | 37.00 | 71.71 |
| Tadjikistan | 36.00 | 69.77 |
| Peru | 32.00 | 62.02 |
| Others | 421.00 | 815.90 |
| World | 7449.00 | 14436.16 |
